# Biosynthesis of Edible Terpenoids: Hosts and Applications

**DOI:** 10.3390/foods14040673

**Published:** 2025-02-17

**Authors:** Mengyu Wang, Zhengyi Zhang, Xinyu Liu, Zhixuan Liu, Ruirui Liu

**Affiliations:** Institute of Environmental Systems Biology, College of Environmental Science and Engineering, Dalian Maritime University, Dalian 116026, China; zhengyi-zhang@dlmu.edu.cn (Z.Z.); dmulxy2022@dlmu.edu.cn (X.L.); swxuan1224@163.com (Z.L.); 15840681379@163.com (R.L.)

**Keywords:** microbial foods, cell factories, terpenoids, biosynthesis, fermentation

## Abstract

Microbial foods include microbial biomass, naturally fermented foods, and heterologously synthesized food ingredients derived from microbial fermentation. Terpenoids, using isoprene as the basic structure, possess various skeletons and functional groups. They exhibit diverse physicochemical properties and physiological activities, such as unique flavor, anti-bacterial, anti-oxidant, anti-cancer, and hypolipemic, making them extensively used in the food industry, such as flavor, fragrance, preservatives, dietary supplements, and medicinal health food. Compared to traditional strategies like direct extraction from natural species and chemical synthesis, microbial cell factories for edible terpenoids have higher titers and yields. They can utilize low-cost raw materials and are easily scaling-up, representing a novel green and sustainable production mode. In this review, we briefly introduce the synthetic pathway of terpenoids and the applications of microbial cell factories producing edible terpenoids. Secondly, we highlight several typical and non-typical microbial chassis in edible terpenoid-producing cell factories. In addition, we reviewed the recent advances of representative terpenoid microbial cell factories with a gram-scale titer in food flavor, food preservation, nutritional enhancers, and medicinal health foods. Finally, we predict the future directions of microbial cell factories for edible terpenoids and their commercialization process.

## 1. Introduction

**Figure 1:**
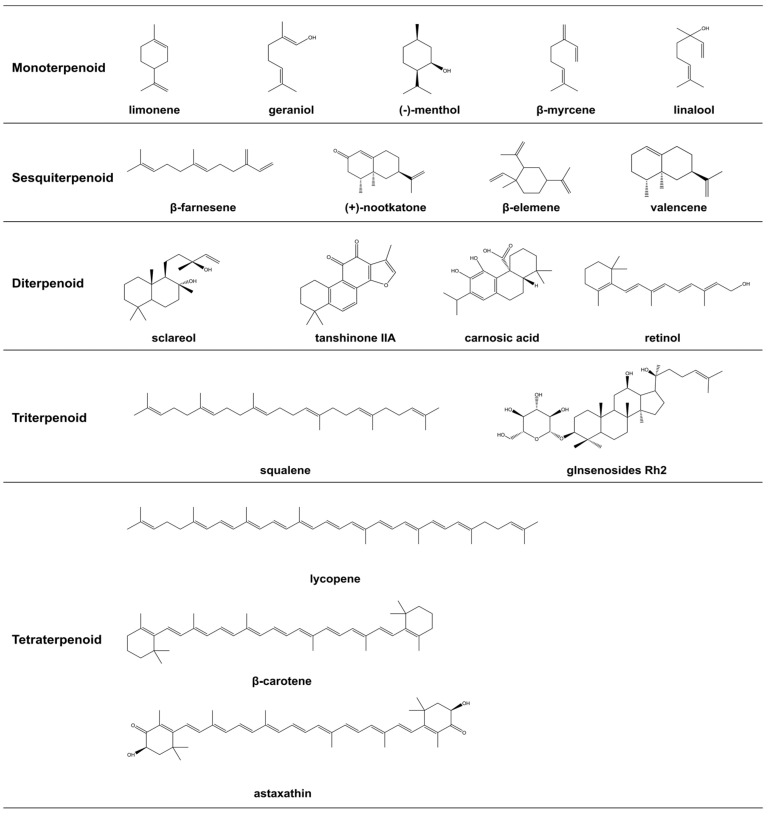
Chemical structures of some terpenoids.

Terpenoids, natural products formed by the head-to-tail linkage of isoprene units, are widely found in organisms, including plants, animals, microorganisms, and marine life. These compounds function as critical secondary metabolites within living organisms. The diversity in their skeleton structures and functional groups confers a broad spectrum of biological activities and physicochemical properties to these compounds, ranging from simple monoterpenes to complex polyterpenes (Figure 1). Terpenoids have substantial application prospects and market demand in fields such as medicine, food, and daily chemicals [1,2,3]. Particularly in the food industry, terpenoid compounds have diverse applications [4]. For example, limonene and menthol are employed as edible fragrances due to their unique flavors and tastes. Carnosic acid and α-pinene exhibit potential as food preservatives owing to their antioxidative and antibacterial properties. Retinol and vitamin K are frequently used as nutritional enhancers in the food industry due to their roles in vision, hemagglutination, and bone metabolism. *Ganoderma* triterpenoids and sclareol are widely used as medicinal health food for their capabilities in hypolipemic and antitumor activities. With the increasing attention to food safety, terpenoids are increasingly applied in the food industry due to their natural origin [5]. Hence, natural terpenoids hold immense potential for application.

However, the low content and uneven distribution of terpenoids in natural plants require the use of complex extraction and separation techniques, resulting in higher extraction costs. Furthermore, their synthesis and accumulation are influenced by various environmental factors such as geographical location and season, making it challenging to produce on a large scale continuously. While chemical synthesis of terpenoids offers scalability, it is encumbered with high raw material costs, intricate processes, and issues related to chiral recognition and stereoselectivity [6]. Microbial cell factories represent an innovative production approach that employs synthetic biology techniques to integrate key enzyme genes of the target biosynthetic pathway into heterologous hosts, such as *Escherichia coli* and *Saccharomyces cerevisiae*. It allows for the direct and efficient production of valuable natural or non-natural chemicals through microbial fermentation, such as amino acids, organic acids, fatty acid derivatives, polyketides, terpenoid compounds, and so on [7]. This production approach has advantages such as a broad spectrum of raw materials, environmentally friendly processing, stereospecific products with high yield potential, and stable year-round production to meet market demands, irrespective of seasons or regions. *Phaffia rhodozyma* naturally produces astaxanthin. Through chemical and ultraviolet radiation, the resulting strain *P. rhodozyma* CBS 6938 achieved an astaxanthin yield 50 times higher than the wild type, thereby reaching commercial levels of production [8]. Meadows et al. redesigned the central carbon metabolism to improve the carbon efficiency and cofactor balance in an engineered farnesene-producing *S. cerevisiae*. The titer of farnesene reached 130 g/L, achieving industrial production [9]. Therefore, the production of edible terpenes using microbial cell factories has become a hot topic in current research [10].

**Figure 2:**
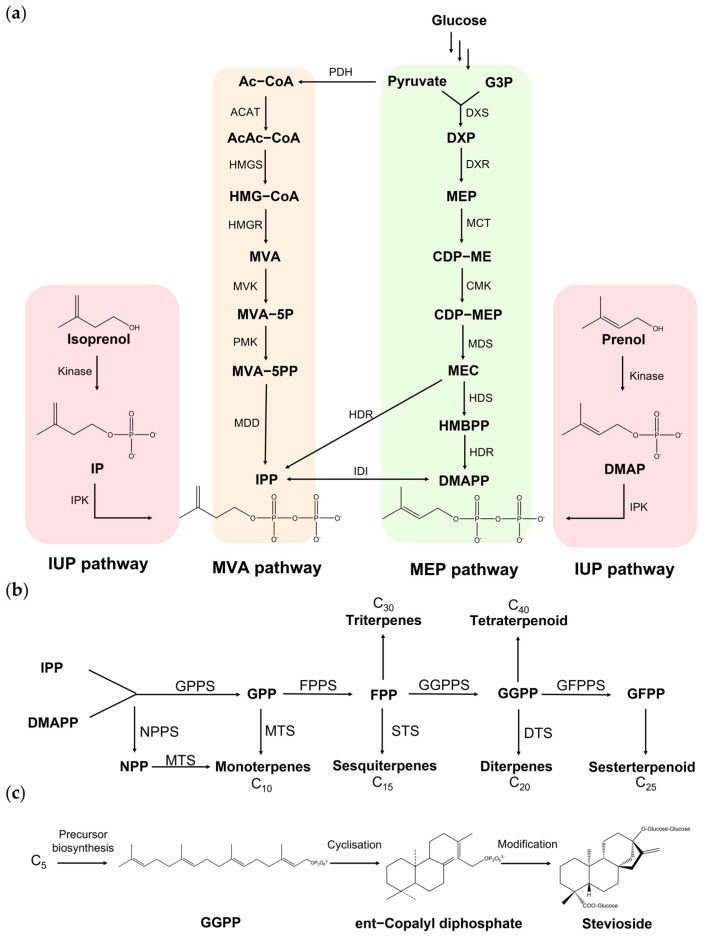
Biosynthetic pathway of terpenoids. (**a**) Natural and non-natural upstream pathways for the C_5_ unit precursors isopentenyl diphosphate (IPP) and dimethylallyl diphosphate (DMAPP). Mevalonate pathway (MVA pathway) in yeast and animal cells utilizes acetyl-CoA (Ac-CoA) as the starting substrate, while methylerythritol 4-phosphate pathway (MEP pathway) in gram-negative bacteria and eukaryotic organelles uses 3-phosphoglycerate (G3P) and pyruvate as the starting substrates. In the non-natural isopentenol utilization pathway (IUP), IPP and DMAPP can be synthesized by sequential phosphorylation steps using isopentenol isomers isoprenol and prenol as precursors. (**b**) Carbon-chain elongation pathway. IPP and DMAPP are condensed to form C_10_, C_15_, C_20_, and other longer terpenoid precursors for monoterpenes, sesquiterpenes, diterpenes, sesterterpenes, triterpenes, and tetraterpenes. (**c**) The biosynthesis of stevioside is presented as an example to demonstrate terpenoid synthesis. C_5_ building blocks condensed to form GGPP as the precursor of diterpenes, then terpene synthases or cyclases helped to form the terpene skeleton, followed by the modification step, such as oxidation and glycosylation.

The biosynthetic pathway of terpenoids is a complex and sophisticated process, including precursor synthesis, skeleton synthesis, and subsequent modification steps [11] (Figure 2). There are two isoprenoid precursor biosynthetic pathways in nature: the mevalonate pathway (MVA pathway), which utilizes acetyl-CoA in the cytoplasm as the starting substrate, and the methylerythritol 4-phosphate pathway (MEP pathway), which uses 3-phosphoglycerate and pyruvate in plastids as the starting substrates (Figure 2a) [12,13]. Through these two pathways, isopentenyl pyrophosphate (IPP) and its isomer dimethylallyl pyrophosphate (DMAPP), which are the basic building blocks of terpenoids, can be synthesized. They condensed in a head-to-tail manner to form monoterpenes (C_10_), sesquiterpenes (C_15_), diterpenes (C_20_), sesterterpenes (C_20_), triterpenes (C_30_), and tetraterpenes (C_40_) (Figure 2b). Subsequently, the bioactive terpenoid compounds can be produced by a series of modification processes, including cyclization, redox reactions, methylation, acylation, and glycosylation (Figure 2c).

Designing and constructing cell factories for high-value terpenoids by utilizing metabolic engineering and synthetic biology strategy, combined with fermentation process control, is gradually demonstrating its unique advantages and potential. Microorganisms serve as key executors in biosynthetic reactions, and their growth physiological characteristics directly influence production efficiency and commercial potential. In this review, we highlight several prokaryotic and eukaryotic microorganisms suitable as chassis for generating edible terpenoids, along with their applications. Subsequently, we present a comprehensive review of the most recent advancements in cell factories for terpenoids utilized in food flavor, preservation, nutritional enhancement, and healthcare. Finally, we provide insights into the prospective directions and challenges associated with the commercial production of edible terpenoids based on microbial cell factories.

## 2. Microbial Chassis as Cell Factories for Edible Terpenoids

Traditionally, terpenoids are extracted from various sources, including plants, animals, microorganisms, and marine organisms [14,15]. However, due to the complex mixture of compounds present in cells, these extracts often contain a diverse array of components. When these extracts or essential oils are used directly as food additives, they may introduce non-target components, potentially leading to unexpected flavors or functions. For instance, steviol glycosides (SGs) extracted from *Stevia rebaudiana* are rich in stevioside, which gives SGs a bitter taste. Furthermore, the yield of active substances obtained from natural materials is often low, failing to meet the high demand. Therefore, chemists continually strive to develop chemical synthesis routes based on terpenoid structures. However, chemical synthesis often fails to fully replicate the intricate configuration of the active ingredients. For example, β-carotene synthesized by chemical methods exhibits an all-trans configuration, which differs from its natural counterpart, and high-dose intake indicates carcinogenic activity [16].

Microorganisms, noted for their rapid reproduction rate, short growth cycle, easily controlled cultivation conditions, mature fermentation processes, and independence from geographical climate influences, have been employed in the synthesis of a variety of compounds. In fact, some microorganisms in nature are capable of producing nutritious and healthy microbial foods via fermentation. However, the variety of terpenoids in wild strains is restricted and insufficient to satisfy diverse human needs. Advancement of omics research has led to the identification of terpenoid pathways and key synthesis enzymes, including cyclases, from various sources and functions. This has provided convenient conditions for the heterologous synthesis of terpenoids [17], thus bringing the microbial synthesis of natural terpenoids into public awareness. Over recent decades, scientists have successfully integrated terpenoid synthesis pathways into both typical and non-typical microbial chassis, achieving the biosynthesis of an array of terpenoids through synthetic biology strategies [18,19].

In 2003, Keasling and colleagues introduced the yeast MVA pathway and amorpha-4,11-diene synthase into *E. coli*, leading to the synthesis of sesquiterpene olefin precursor to artemisinin, namely amorphadiene [20]. A decade later, the cytochrome *b*_5_ cDNA sequence and dehydrogenase gene were introduced into yeast, resulting in a high-yield engineered strain for producing artemisinic acid, a precursor of artemisinin. This patented technology was subsequently employed by Sanofi to produce tens of tons of artemisinic acid annually. When combined with chemical methods, this achievement becomes a significant milestone in the successful application of synthetic biology [21]. In terms of isoprene, Martos assessed various factors, including materials, energy efficiency, economic feasibility, and land use, and revealed that the *E. coli* cell factory is economically viable compared to conventional petrochemical alternatives [22].

### 2.1. Prokaryotic Microorganisms

Prokaryotic microbial cells, characterized by their simple cellular structures, rapid reproduction, robustness, ease of genetic manipulation, and metabolic regulation, are frequently used in the construction of cell factories. Most prokaryotic microorganisms, including gram-negative bacteria and cyanobacteria, possess an endogenous MEP pathway capable of producing IPP and DMAPP [13]. A stoichiometric and redox balance analysis reveals that the MEP pathway exhibits high carbon efficiency. For each molecule of IPP produced, the pathway consumed 1.196 molecules of glucose, representing a carbon consumption 14% higher than the MVA pathway [23]. Consequently, prokaryotic microorganisms are extensively employed for the synthesis of terpenoid compounds (Table 1).

#### 2.1.1. *E. coli*

*E. coli*, possessing a well-defined genetic background and comprehensive genetic manipulation techniques, serves as a model prokaryotic microorganism. Presently, diverse strategies have been employed to enhance the titer of terpenoids in *E. coli* [41].

In 2009, Church et al. optimized and combined the RBS of 20 endogenous genes in *E. coli* to enhance the flux of the DXP pathway and achieved a 5-fold increase in lycopene production [24]. The multivariate-modular approach to metabolic-pathway engineering strategy was used to optimize the endogenous MEP pathway for IPP synthesis and the heterologous pathway for taxadiene synthesis, achieving a 15,000-fold increase in taxadiene production [23]. Nevertheless, dependence solely on the endogenous MEP pathway remains suboptimal. A study reported that by reconstituting five genes in the MVA pathway and the *idi* gene in *E. coli*, a three-fold increase in lycopene titer was realized [42]. In recent years, several naturally occurring plant-derived terpenoids have also been biosynthesized in *E. coli*.

In Li’s lab, they screened for an active geranyl diphosphate synthetase (GGPS) and fused a truncated leader peptide to promote the soluble expression of geraniol synthetase (GES). The geraniol titer of engineered *E. coli* increased to 2.12 g/L [25]. In order to alleviate the toxicity of the end product, they introduced an alcohol acyltransferase to esterify geraniol to geranyl acetate, and then they used another *E. coli* expressing acetyl esterase, which hydrolyzes geranyl acetate to geraniol. By fed-batch fermentation in a 10 L bioreactor, the titer of geraniol was reached to 13.19 g/L [26]. Further, they recruited a truncated linalool dehydratase isomerase (LDI) to isomerize geraniol to linalool and achieve de novo biosynthesis of myrcene at 1.25 g/L by using aerobic-anaerobic two-stage fermentation [27]. In order to efficiently produce plant-derived sesquiterpenes β-elemene in *E. coli*, Bai and his colleagues systematically designed the metabolic pathway. They screened for a germacrene A synthase (NS), which could transform farnesyl pyrophosphate (FPP) to β-elemene via germacrene A, engineered the RBS of NS, and linked NS with farnesyl diphosphate synthase (IspA). After rewriting the native central carbon metabolism of acetyl-CoA and pyruvate and overexpressing two membrane transporters, TolC and MsbA, to pump compounds extracellularly, they achieved the highest reported titer of 3.52 g/L of β-elemene and 2.13 g/L of germacrene A [28].

*E. coli* cell factories can not only elongate carbon chains but also cleave carbon chains. As we know, carotenoid cleavage dioxygenases (CCDs) could catalyze the cleavage and oxidation of α-carotene or ε-carotene to form ionone. Huang et al. used an evolved thioredoxin-CCD1 fusion protein in a lycopene-producing strain to synthesize α-ionone. However, they found that the by-product H_2_O_2_ of CCD1 will break down the precursor lycopene. So, they overexpressed the alkyl hydroperoxide reductase (*ahp*C/F) to reduce oxidative stress. As a result, they achieved 700 mg/L α-ionone in a 5 L bioreactor [29]. For dihydro-β-ionone, Zhao and his colleagues identified an enoate reductase (DBR1) gene from *Artemisia annua*, which can transform β-ionone to dihydro-β-ionone. Then, they constructed a cell-free system containing CCD1, DBR1, and glucose dehydrogenase (GDH) for dihydro-β-ionone production. The molar conversion was 85.8% [30]. Furthermore, they constructed a de nove β-ionone biosynthesis platform in *E. coli*. When co-cultured with *S. cerevisiae* harboring DBR1, the final yield of dihydro-β-ionone was achieved at 27 mg/L [31].

#### 2.1.2. *Corynebacterium glutamicum*

*C. glutamicum*, well-known for rapid growth, broad substrate spectrum, and resistance to bacteriophage infections, and can be cultivated on a large scale, is generally regarded as a safe (GRAS) organism [43,44]. Therefore, it is often used in the food industry, particularly in the large-scale production of amino acids [45]. With the improvement of genetic manipulation techniques, its production spectrum has expanded, and it is often developed into a cellular factory for terpenoids.

*C. glutamicum* contains glycosylated C_50_ carotenoids decapreoxanthin, which is a yellow pigment. It indicated that *C. glutamicum* has an abundance of FPP and GGPP precursors. In order to extend the ability to synthesize other carotenoids, Choi et al. integrated their newly identified gene cluster *crtNaNcM* from *Planococcus martimus* into the *C. glutamicum* genome. Various novel C_35_ carotenoids were detected in the cells. It indicates that *C. glutamicum* can serve as a promising carotenoid cell factory [32]. Li et al. knocked out the key genes involved in the native C_50_ carotenoid synthesis, ranging from *crtE* to *crtYe*, and strengthened the expression of CrtE/B/I to increase the lycopene level. Additionally, they discovered a bifunctional enzyme, OluLCY, exhibiting the highest ε-cyclase activity and the lowest β-cyclase activity, and performed multi-copy integration of *oluLCY* to the host. By further strengthening the MVA pathway to increase the supply of IPP, they obtained a strain with an α-carotene titer of 1.054 g/L. It was the first report of α-carotene synthesis in *C. glutamicum* [33]. Henke and co-workers assembled lycopene β-cyclase, β-carotene hydroxylase, and β-carotene ketolase in a lycopene-producing *C. glutamicum* platform to produce astaxanthin via β-carotene. By optimizing and balancing the expression of CrtZ and CrtW, an astaxanthin titer of 103 mg/L was achieved in a 2 L fed-batch bioprocess. Furthermore, the pathway was expanded towards astaxanthin-β-d-diglucoside biosynthesis by additional expression of a glycosyltransferase CrtX [34].

*C. glutamicum* is a non-ubiquinone-containing bacterium. To produce Coenzyme Q_10_, Burgardt et al. increased the supply of precursor FPP and decaprenyl diphosphate (DPP) by expressing FPP synthase (IspA) and DPP synthase (DdsA). Then, they knocked out the side-product synthesis pathway and replaced the key synthetic enzyme with a mutant that eliminates feedback inhibition, thereby increasing the flux into the shikimic acid pathway to synthesize pHBA. After they enhanced the precursor supply pathway, the 10 HB condensation pathway, and subsequent modification pathways into *C. glutamicum*, the resultant strain UBI413 was shown to synthesize CoQ_10_ [35].

#### 2.1.3. *Bacillus subtilis*

*B. subtilis*, a model strain of gram-positive bacteria, possesses clear genetic information. It can be easily manipulated genetically and shows a strong ability to utilize inexpensive substrates. Since it has a fast growth rate and robust expression and secretion capabilities, it is extensively used in the production of chemicals and protein products. In addition, it has an endogenous MEP synthesis pathway and is considered GRAS [46]. Thus, it is a potential chassis for edible terpenoids.

The introduction of heterologous MVA pathways in bacteria presents an effective strategy for enhancing IPP supply. However, the static regulatory strategies may potentially impact the titer and yield of the cell factories because of the accumulation of toxic intermediates and metabolic imbalances. To address this, researchers have devised a dynamic regulation strategy aimed at balancing the metabolic flux between the MEP and MVA pathways in *B. subtilis*. In this research, a series of genetic circuits activated by pyruvate and inhibited by malonyl-CoA were designed to respond to intracellular levels of pyruvate and malonyl-CoA, and an AND gate was used to balance the flux of the MEP and MVA pathways to reshape the IPP synthesis pathway. In the strain, BS17-P20 synthesizing menaquinone-7 (MK-7, Vitamin K2-7) with dynamically regulated central metabolic pathways, IPP synthesis pathways, and terpenoid synthesis pathways, the level of IPP was increased by 4-fold, and the MK-7 titer reached 467.2 mg/L. This dynamic cascade regulation system is also applicable to the biosynthesis of β-carotene; the titer of carotenoids and lycopene in the engineered bacteria BS17-P13 increased by 1.9 and 10 times, respectively [36]. In another case, all the genes in the MEP pathway were integrated on a plasmid to increase the flux of the MEP pathway. The precursor FPP supply was increased by enhancing the expression of IspA. Then, GFP was fused to the N-terminus of the amorphadiene synthase (ADS) to improve its translation efficiency. These metabolic strategies combined with medium optimization enabled *Bacillus subtilis* to synthesize amorphadiene with a yield of 416 mg/L [37].

#### 2.1.4. *Cyanobacteria*

Cyanobacteria are a kind of photosynthetic autotrophic microorganism that can grow using sunlight and trace minerals without an additional carbon source. Due to their endogenous MEP pathway, cyanobacteria have been recognized as novel promising hosts as a large-scale platform for light-driven terpenoid biosynthesis [47,48].

There are three model cyanobacteria, *Synechococcus elongatus* PCC 7942, *Synechococcus* sp. PCC 7002, and *Synechocystis* sp. PCC 6803 [38]. Scientists compared the potential of two models, *S.* PCC 7002 and *S.* PCC 7942, in producing terpenes. They expressed the corresponding terpene synthase by the same strong promoter in the plasmid into the two hosts and measured their terpene yield. It was found that different chassis have different product spectrums. It is suggested that the choice of host is a crucial step when designing and constructing synthesizing terpene cell factories [49]. Using *S.* PCC 7942 as the host, it only requires the expression of codon-optimized cineole synthase (CnsA) to directly convert intracellular GPP into 1,8-cineole [38]. Similarly, when the gene of pinene synthase originated from *Abies grandis* (*Ag*PS) was integrated into the chromosome, the pinene titer of *S.* PCC 7002 was achieved at 1.525 mg/L under the condition of CO_2_ aeration and continuous illumination [39]. In *S.* PCC 6803, Dietsch et al. knocked out squalene synthase and squalene hopene cyclase coding sequences for the FPP branch pathway to avoid squalene by-products. Furthermore, they knocked down the key gene *crtE* in the GGPP derivative pathway and introduced a heterologous FPP synthase (IspA) to increase the supply of precursor FPP. In order to further improve the conversion rate, they fused IspA and valencene synthase (*Cn*VS) for co-expression. The resulting strain produced 19 mg/g DCW of valencene [40]. Unlike other work producing terpenoids using traditional enzyme engineering and pathway engineering strategies, Rodrigues and co-workers focused on optimizing cultivation conditions in the isoprene production in cyanobacteria. They found that the yield of isoprene increased under violet light and elevated temperature. This provides a new strategy for other cyanobacteria-based cell factories to produce terpenoids [50].

### 2.2. Eukaryotic Microorganisms

In eukaryotic microorganisms, the MVA pathway is used to synthesize terpenoid precursors IPP and DMAPP. Compared with prokaryotic microorganisms, the structure of eukaryotic chassis is more complex, providing a necessary place for compartmentalized metabolic regulation and facilitating the expression of transmembrane proteins. The post-translational modification capabilities in eukaryotic cells are beneficial for the correct expression and function of enzymes derived from plants and other eukaryotic cells. In addition, eukaryotic microorganisms have strong adaptability and tolerance to environmental conditions, making them more stable and efficient in specific industrial environments (Table 2).

#### 2.2.1. *S. cerevisiae*

*S. cerevisiae* is a GRAS species with a clear genetic background and metabolic pathway, efficient tools for gene manipulation, and metabolic regulation, making it the most popular chassis in microbial cell factories. It has been used for the production of monoterpenes, diterpenes, triterpenes, and even more complex terpenoids, such as menthol, sclareol, squalene, and so on [73,74,75].

Shu et al. identified a β-myrcene synthase (MS) t*Qi*MS from six plant-derived sources that can induce *S. cerevisiae* to produce myrcene. Combined with rational design, reverse fusion with ERG20^WW^, and peroxisome localization, a β-myrcene titer of 142.64 mg/L was obtained in a 5 L bioreactor [51]. Ma et al. discovered a (-)-bornyl diphosphate synthase *Bb*TPS3 based on transcriptome. With further codon optimization and fusion expression with ERG20^F96W-N127W^, a (-)-borneol titer of 148.59 mg/L in a 5 L bioreactor was achieved [52]. In the case of retinol, Hu et al. integrated the β-carotene dioxygenase (BLH) gene into the previously constructed β-carotene engineering strain to constitute a complete vitamin A synthesis pathway. They further increased the supply of precursor β-carotene and cofactor NADPH through metabolic engineering. Additionally, two reductases, Env9 and YbbO, were introduced into the strain to further enhance retinol production. To reduce the oxidation of the produced retinol, butylated hydroxytoluene (BHT) antioxidant was added during high-density fermentation, ultimately yielding 2479.34 mg/L of retinol [53]. For the more complex phenolic tricyclic diterpene carnosic acid (CA), scientists not only overexpressed *Sm*CPS and *Sm*KSL to provide the terpene skeleton miltiradiene but also expressed the related modifying enzymes CYP76AK6, CYP76H24, and CYP76AH1 to synthesize carnosic acid. In order to promote electron transportation and release oxidative stress, they expressed cytochrome P450 reductase and NADPH-cytochrome B5 fusion protein (*Sm*CPR~t28*Sp*Cytb5) and *Sc*CTT1 catalase. Finally, by enhancing the supply of NADPH and heme and activating the transcription of proteins folding-related genes in the endoplasmic reticulum, they obtained the best strain, WCA11, with a CA titer of 75.18 mg/L [54].

The natural isoprenoid biosynthesis pathway is often limited by low carbon and energy efficiency. Researchers have designed a novel pathway, namely the isopentenol utilization pathway (IUP), in *E. coli*, which can generate DMAPP and IPP through sequential phosphorylation of isoprenol or prenol (Figure 2a) [76]. It was reported that the advantage of the IUP is its minimal cofactor requirement, where only two molecules of ATP are needed to synthesize one molecule of IPP. However, it was found that isopentenol in the IUP pathway inhibits the respiration of *S. cerevisiae*. In order to overcome this problem, Li et al. designed an IU pathway-dependent (IUPD) strain with growth-coupled production. By utilizing the auxotroph phenotype towards isoprenol, they developed a growth-coupled high-throughput screening method and identified two kinase mutants with higher activity, *Sm*DAGK^S47A/L124A^ and *At*IPK^S270P/A272R^. The resultant strains with strengthened IUP pathways had higher fluxes of IPP/DMAPP, producing more squalene, limonene, and carotenoids [77].

#### 2.2.2. *Yarrowia lipolytica*

In order to avoid the overflow of carbon metabolism and generation of by-product ethanol, scientists have focused their attention on Crabtree-negative species. *Y. lipolytica*, as a model strain of oleaginous yeasts, is Crabtree-negative and GRAS [78]. It possesses high levels of the tricarboxylic acid (TCA) cycle and the pentose phosphate (PP) pathway. Through these two pathways, a large amount of acetyl-CoA, ATP, and NADPH can be accumulated, making it a promising and superior chassis for terpenoid compounds [79].

The level of precursors IPP and DMAPP determines the yield of subsequent metabolites. To increase the precursor level, key enzymes CHK and IPK in the IUP pathway were introduced into *Y. lipolytica*, resulting in a 15.7-fold increase in IPP and DMAPP. By overexpressing ACC1 and DGA1 to promote lipid accumulation, the intracellular hydrophobicity was modulated, thereby increasing the storage capacity for lycopene. With exogenous palmitic acid supplementation, the degradation blocks acetyl-CoA from β-oxidation further enhanced the natural MVA pathway. The final lycopene titer was 4.2 g/L in 3 L batch bioreactors [55]. This indicates that the hydrophobic environment of oleaginous yeast is beneficial for the accumulation of terpenoids. In geraniol production, Agrawal et al. expressed the truncated plant-derived geraniol synthase (t*Cr*GES) in *Y. lipolytica* and then integrated ERG10, HMGS, tHMG1, and IDI into the chromosome to enhance precursor GPP supply. The resulting strain S5 produced geraniol with a titer of 1 g/L shake-flask fermentation [56]. Sun et al. co-expressed the key enzymes t*Ss*LPPs and *Ss*SCS of sclareol synthesis pathway in *Y. lipolytica* with tHMG1 and ERG20 overexpression, enabling the host to synthesize sclareol. They further designed a two-layer GGPP accumulation pathway, introducing *Pa*GGPPS and *Ss*GGPPS to simultaneously utilize IPP, DMAPP, and FPP to increase GGPP supply. Then, by using protein scaffold peptide tags RIAD and RIDD, they assembled t*Ss*LPPS and *Ss*SCS into multi-enzyme complexes to facilitate substrate transport and reduce the production of by-products geranylgeraniol (GGOH) and labdendiol (LOH). The engineered strain produced sclareol as much as 12.9 g/L [57]. To facilitate genetic modification in *Y. lipolytica*, Xu and co-workers knocked out the non-homologous end joining protein Ku70 and co-expressed the key homologous recombination proteins RAD52 and RAD59, achieving a homologous recombination efficiency of 68% without affecting the growth. They also systematically reshaped metabolism by enhancing MVA pathway flux, increasing the supply of acetyl-CoA and NADPH, and weakening the pathway for squalene degradation to ergosterol. In a 5 L fed-batch fermentation, the engineered strain achieved a squalene titer of 35 g/L [58]. In another case, Shi et al. transformed a carotenoid cleavage dioxygenase (*Of*CCD1) with a strong multi-round promoter iteratively into *Y. lipolytica* to construct a β-ionone-producing strain. Then, they further broaden the substrate range by overexpressing the key enzymes for xylose metabolism, xylose reductase (*Ss*XR), xylitol dehydrogenase (*Ss*XDH), and endogenous xylulose kinase (XK). The final engineered strain could synthesize β-ionone using lignocellulosic hydrolysate as the carbon source [59]. It indicates that *Y. lipolytica* has the potential to utilize low-cost raw materials for the green production of high-value terpenoids.

#### 2.2.3. Methylotrophic Yeasts

Methylotrophic microorganisms can utilize one-carbon compounds as a carbon source for growth. *Ogataea polymorpha* and *Pichia pastoris*, which are Crabtree-negative, have been receiving wide attention as hosts due to their ability to use methanol as a carbon and energy source. They possess high protein expression and secretion capabilities and allow for high-density fermentation [80]. The properties make them beneficial for biomanufacturing processes and widely used for efficient protein production. Since they are GRAS species, enzymes produced by these two yeasts, such as lipases, have been used as enzyme preparations in the food industry [81] already. Their robust five-carbon assimilation capacity makes them potential hosts to use lignocellulosic hydrolysates as raw materials. In addition, their endogenous MVA pathway can provide abundant precursors IPP and DMAPP for terpenoids, indicating that *O. polymorpha* and *P. pastoris* hold significant potential as cell factories for terpenoids [80].

Li et al. screened a farnesene synthase (*Aa*FS) with high activity and introduced it into the fatty acid-producing *O. polymorpha* strain M16 in their previous research. By further increasing the flux of the MVA pathway and the supply of precursor acetyl-CoA, the optimized engineering strain produced β-farnesene with a yield of 46 mg/g methanol, which is the highest reported production from one carbon [69]. Ye and co-authors identified an efficient germacrene A synthase, *Ls*LTC2, in *O. polymorpha*. Furthermore, they enhance the acetyl-CoA, MVA, and NADPH flux and downregulate the sterol competition flux. Through spontaneous Cope rearrangement from germacrene A. They obtained β-elemene at a titer of 4.7 g/L in the fed-batch fermentation [61].

In another methylotrophic yeast, *P. pastoris*, researchers have successfully constructed cell factories capable of producing terpenoid compounds such as germacrene A, santalene, and lycopene by introducing corresponding terpene synthases and performing conventional metabolic engineering [62,63,64]. In the case of producing α-farnesene, Liu et al. adopted the heterologous IUP pathway and a peroxisome-cytoplasm dual regulation strategy to enhance the α-farnesene synthetic flux. Firstly, they screened and utilized a high-activity farnesene synthase (*Md*AFS) and strengthened the MVA and acetyl-CoA pathway. Secondly, they targeted the more efficient IUP pathway to peroxisomes to promote the accumulation of IPP and DMAPP. Finally, by simultaneously regulating the IPP and α-farnesene synthetic pathway in the cytoplasm and peroxisomes, they obtained 1.4 g/L of α-farnesene in the medium co-feeding with oleic acid and sorbitol as carbon sources [65]. Peroxisome compartmentalization not only alleviates the burden on growth caused by the accumulation of cytotoxic intermediates such as IPP but also separates IPP from competitive pathways in the cytoplasm and has now been used for the construction of various cell factories. In another case, *P. pastoris* has been used to synthesize the precursor of a more complex ginsenoside precursor, dammarenediol-II. To facilitate the transfer of substrates and alleviate the interference from competing ergosterol pathway, researchers expressed squalene epoxidase (ERG1) and dammarenediol synthase (*Pg*PDD) separately using PDZ domain and its corresponding ligand (PDZlig), respectively. Ultimately, the yield of dammarenediol-II in the self-assembling multi-enzyme complex strain was 2.1-fold higher than that of the free expression strain [66].

#### 2.2.4. *Rhodosporidium toruloides*

*R. toruloides* is a famous lipid yeast that can accumulate a large amount of lipids under nutrient-limited conditions. Its lipid level exceeds 70% of the dry cell weight [82], and its red phenotype is related to the natural ability to synthesize carotenoids. The high levels of lipids and carotenoids indicate that *R. toruloides* has sufficient acetyl-CoA precursors, making it an ideal candidate for terpenoid cell factories. In addition, it also has advantages such as high-density growth, excellent stress resistance, and a wide substrate spectrum, allowing for large-scale fermentation using inexpensive raw materials [83]. Although it does not possess an efficient endogenous homologous recombination system like model microorganisms such as *S. cerevisiae*, lots of gene manipulation tools have been developed recently [84], enhancing the convenience and efficiency of homologous recombination significantly. Currently, different terpenoid compounds have been synthesized by *R. toruloides*. Apart from *R. toruloides*, other oleaginous red yeasts have also been modified to synthesize terpenoids successfully [85,86].

In order to directly utilize the excellent performance of *R. toruloides* in accumulating carotenoids, Tran et al. obtained a mutant strain, G17, with increased astaxanthin content by using gamma irradiation mutagenesis. They used response surface methodology to optimize the large-scale culture conditions, enabling *R. toruloides* to produce astaxanthin with a content reaching 1.26 mg/L using molasses as a carbon source [67]. In the case of limonene, Yang and co-workers introduced an orthogonal limonene synthesis pathway based on neryl pyrophosphate (NPP) into *R. toruloides*. They used carotenoid-deficient *R. toruloides* as the host to block competing pathways, enhanced the MVA pathway flux, and simultaneously expressed the key enzyme neryl pyrophosphate synthase and limonene synthase for the downstream limonene synthesis pathway. The engineered strain achieved a final limonene yield of 393.5 mg/L [68]. Furthermore, they identified several peroxisomal targeting signals (PTSs) and used them to target MVA and limonene biosynthetic pathways to peroxisomes. Then, they carried out dual-metabolic regulation in the cytoplasm and peroxisome to enhance the supply of acetyl-CoA. A higher yield of limonene was finally reached at 1.05 g/L [69]. Similarly, Geiselman et al. utilized metabolic engineering and Design-Build-Test-Learn (DBTL) strategies to introduce the ent-kaurene biosynthesis pathway and precursor GGPP supplying pathway into *R. toruloides*. The resultant strain obtained ent-kaurene at a titer of 1.4 g/L in a 2 L bioreactor feeding corn stover hydrolysates [70]. In order to further expand the application of *R. toruloides* in the production of terpenoid compounds using corn stover hydrolysate, Gladden and colleagues introduced bisabolene synthase (BIS) and amorphadiene synthase (ADS) into *R. toruloides* to produce bisabolol and amorphadiene, and also introduced GPPS and 1,8-cineole synthase to produce 1,8-cineole. Through metabolomic and proteomic analysis, they identified the rate-limiting steps and balanced the MVA pathway and downstream pathway. Finally, they obtained strains for bisabolene, amorphadiene, and 1,8-cineole with titers of 36 mg/L, 2.6 g/L, and 1.4 g/L, respectively [71,72].

## 3. Application of Terpenoid Microbial Cell Factories in the Food Industry

Terpenoids have complex and diverse structures, with various functional groups and active sites, thus exhibiting different biological activities, such as special fragrances, antioxidant, antibacterial, and anticancer effects. Moreover, due to the complexity of terpenoid structures, a single terpenoid compound can play more than one role in a food product. For instance, squalene acts as both an antioxidant and an antimicrobial agent in muffin preservation [87]. In the food industry, terpenoids are highly favored due to their natural source. However, extraction and purification from natural plants or animals is cumbersome and inefficient. Nowadays, an increasing number of microbial hosts have been engineered for the biosynthesis of terpenoids, and some terpenoids have achieved gram-scale production in microorganisms. Here, we provide an overview of several representative terpenoid-producing microbial cell factories that have been used in the food industry (Table 3).

### 3.1. Acting as Flavor and Fragrance

Terpenoids, which are in forms of esters, alcohols, and oxides, are commonly found in fruits, vegetables, and nuts. Different terpenes have different fragrances. They are the main components of essential oils and are widely used as natural fragrances and flavors. Linalool, geraniol, nerolidol, and other monoterpenes and sesquiterpenes have been recognized as GRAS by the United States Food and Drug Administration (FDA) [106]. In the food industry, they have been used as food additives to provide food with a unique fragrance and taste [11]. For example, monoterpenes such as menthol and limonene are often used as flavor additives in foods like chewing gum, beverages, and candies. The boosting strategies of metabolic engineering and synthetic biology in cell factories have greatly promoted the sustainable production of terpenoid compounds [107,108].

#### 3.1.1. Linalool

Linalool is an acyclic monoterpene and is widely used in beverages and processed foods due to its flavoring and fragrant properties.

It has two enantiomers, (S)-linalool and (R)-linalool. The two forms of linalool have different fragrance profiles and biological properties, while separation and purification are difficult when extracting from plants. Hoshino et al. identified (S)-specific linalool synthase (LINS) and (R)-specific LINS and co-expressed them with mutated farnesyl diphosphate synthase (IspA*) in *Pantoea ananatis* host. The titer of (S)-linalool and (R)-linalool reached 5.6 g/L and 3.71 g/L, respectively [91]. In order to improve the catalytic activity for linalool synthase, Zhou et al. semi-rationally designed its substrate binding pocket entrance and obtained a mutant t67OMcLIS_M_^F447E^ with higher affinity for GPP and higher catalytic activity to synthesize linalool. Furthermore, they targeted the linalool biosynthetic pathway to *S. cerevisiae* peroxisomes and obtained a more stable diploid engineering strain through mating. The resultant strain achieved a titer of 2.6 g/L linalool in a 5 L fed-batch fermentation [89]. In another research, Wang and co-workers introduced linalool dehydratase, which can isomerize geraniol to linalool and subsequently dehydrate linalool to myrcene, into *E. coli*. The engineered strain produced myrcene at a titer of 1.25 g/L in aerobic and anaerobic two-stage fermentation [27].

#### 3.1.2. Geraniol

Geraniol is an acyclic monoterpene alcohol with a rose-like odor and a sweet, floral, rose-like taste. It is an important raw material in the commercial fragrance industry. It is reported that various microorganisms can convert geraniol into high-value terpenoid products such as (R)-citronellol, (S)-citronellol, nerol, and neral [109]. Besides its unique scent, it also possesses biological activities like anti-microbial, anti-cancer, anti-oxidant, and anti-inflammatory. Therefore, geraniol is widely used in fields such as food, cosmetics, and pharmaceuticals, exhibiting great commercial value.

Nowadays, researchers have identified that geraniol synthase (GES) originated from various plants and assembled a geraniol de novo synthetic pathway in microorganisms, such as *E. coli* [26], *S. cerevisiae* [110], *Y. lipolytica* [56], and *Candida glycerinogenes* [111]. However, the toxicity of geraniol to cells limits the production of microbial cell factories. Conversion of geraniol to its acetate form is an effective method to reduce its toxicity. Shukal and co-workers combined metabolic engineering, enzyme engineering, ribosomal binding site, and abiotic engineering in *E. coli* to produce geranyl acetate by using glycerol as feeding stock. To circumvent potential issues of insufficient solubility or low activity of GES, they utilized nucleoside diphosphate-x (NUDIX) instead of GES to produce geraniol. After regulating the MVA pathway flux, they fusion-expressed the efficient GPPS and NUDIX for geraniol synthesis. Then, they knocked out alcohol dehydrogenases and adopted microaerobic cultivation to avoid the conversion of geraniol to geranial. Finally, by introducing the alcohol acyltransferase *Rh*AAT1, the strain transformed geraniol into the low-toxicity geranyl acetate. The final yield of geranyl acetate reached the highest reported level of 19 g/L [90].

### 3.2. Acting as Biopreservatives

With the increasing awareness of the potential health risks posed by food additives, the concept of clean-label foods gained more attention. Natural preservatives not only extend the shelf life of food but also offer additional health benefits [112]. It was reported that the global sales of natural food preservatives are estimated to be 612 million by 2024, and it might increase up to 2-fold ten years later (https://www.futuremarketinsights.com/reports/natural-food-preservatives-market (accessed on 13 February 2025)).

Due to the hydrophobic skeleton, terpenoids are often lipophilic so they can disrupt cell membranes and inhibit enzymes related to energy metabolism and DNA synthesis, leading to impaired cell viability [5]. For instance, α-pinene and limonene can inhibit the activity of *E. coli* and *Staphylococcus aureus*. On the other hand, some terpenoids act as antioxidants by scavenging free radicals and protecting food from oxidative damage [14]. For example, carotenoids are widely used as antioxidants due to their conjugated double-bond system. In this scene, terpenoids with antibacterial and antioxidant bioactivities have great potential as food preservatives [113]. In fact, terpenoids have already been commonly used as food preservatives in fruits, meat products, seafood, and dairy products, in the form of natural extracts or essential oils [112,114]. However, since extracts and essential oils contain multiple components, achieving their minimum inhibitory concentration (MIC) often requires high-level supplementation. Additional chemicals might affect the taste of the food or introduce undesirable flavors [112]. Terpenoid cell factories through metabolic engineering may meet demands for food preservation and food safety.

#### 3.2.1. Limonene

Limonene is a natural monoterpene with antibacterial efficacy and has been widely used in the food industry as a preservative, antioxidant, and flavor. Its derivatives, such as menthol, carveol, and α-terpineol, which have pleasant fragrances, special biological activities, and physicochemical properties, are also broadly used in the fields of food, pharmaceuticals, and cosmetics [115]. Cell factories offer a new approach to the sustainable production of limonene.

In *S. cerevisiae*, Kong et al. expressed truncated limonene synthase (tLimS) to de novo synthesize limonene from GPP. They further enhanced the limonene synthesis flux through a “Push−Pull−Restrain” strategy while simultaneously increasing the supply of acetyl-CoA and NADPH by regulating the glyoxylate cycle and pentose phosphate pathway. In addition, they reconstructed the MVA and limonene synthetic pathways in mitochondria. In order to alleviate the impact of limonene accumulation on cell growth, they also performed a two-phase scale-up fermentation and obtained a limonene titer of 2.63 g/L ultimately [91]. It appears that the titer of limonene in most cell factories has reached the cells’ maximum tolerance to limonene. In order to enhance the tolerance of *Y. lipolytica* to limonene, Kong et al. identified five genes using a transcriptomic analysis. They found that overexpression of an unknown protein YALI0F19492p can increase limonene tolerance and increase the titer up to 8-fold. Then, they carried out short-term laboratory adaptive evolution and obtained strains with higher tolerance. Field emission scanning electron microscopy (FE-SEM) and propidium iodide (PI) analyses revealed that the cell wall and membrane of evolved strains were intact in the presence of limonene, while both the cell wall and membrane of the starting strain were damaged [92]. It would provide new perspectives for improving the sustainable production of limonene.

#### 3.2.2. Pinene

Pinene is a monoterpenoid derived from plants, and it is widely used as flavors, fragrances, and pharmaceuticals. Its dimerized form can serve as a high-density renewable fuel for aircraft [39]. Moreover, pinene can also be used as a raw material for the synthesis of fragrances such as terpineol and linalool.

In the monoterpene cell factories, only one heterologous monoterpene synthase is usually needed for the production of monoterpenes. In *E. coli*, Zhou et al. identified an effective pinene synthase *Pt*PS1^Q457L^ and improved its hydrophilicity and solubility by N-terminal truncation, facilitating folding and preventing degradation. Next, they introduced a GPPS mutant *Ag*GPPS^D90G/L175P^ to enhance the supply of precursor GPP and optimize fermentation conditions. Ultimately, in the optimal host DH10B, a titer of 1035 mg/L pinene was produced in 1.3 L fed-batch fermentation [93]. In order to break through the limitations of mass transfer and reduce the impact of pinene on cell viability, Niu et al. achieved gram-scale production of pinene using a cell-free system. They constructed a module co-catalysis cell-free system containing an MEV upstream module and a pinene downstream module and optimized the physicochemical parameters using Plackett-Burman experimental design and the steepest ascent strategy. The final pinene titer was 1256 mg/L [94]. To some extent, cell-free systems are an effective alternative to whole-cell catalysis for synthesizing terpenoids with toxicity.

### 3.3. Acting as Food Fortifiers and Dietary Supplements

Terpenoids also have other beneficial biological effects besides flavoring and anti-bacterial properties. They can be used as dietary supplements to enhance the nutritional value of food and improve the health level of people. Retinol converts into retinal and retinoic acid, which are involved in the synthesis of rhodopsin on the retina [116]. Lutein plays a crucial role in protecting the retina and preventing age-related macular degeneration [117]. As functional ingredients, they are often added to infant formula foods, edible oils, and beverages. Anthocyanin is also a famous dietary supplement due to its antioxidative and hypoglycemic effects [118]. Therefore, designing and constructing microbial cell factories for functional terpenoid compounds has great application potential.

#### 3.3.1. Lycopene

Lycopene is a red C_40_ tetraterpene, consisting of eight isoprene units (C_5_) linked head-to-tail. It is capable of quenching singlet oxygen and scavenging free radicals to prevent some malignancies and support cardiovascular health [119]. It was reported that lycopene exhibits the highest antioxidant activity among the carotenoid group [120]. Thus, it is widely used as a colorant and functional food. However, the chemical synthesis of tetraterpenes faces difficulties in stereoselectivity and separation [120]. Therefore, many attempts have been made to construct sustainable production cell factories for lycopene.

In nature, some microorganisms, such as *R. toruloides* and *Blakeslea trispora*, possess carotenoid biosynthesis pathways and can synthesize mixtures of carotenoids, including lycopene, β-carotene, and astaxanthin. Traditionally, these microorganisms can be directly used for the fermentative production of lycopene. However, it is very difficult to improve the yield and purity of a particular kind of carotenoid. With the advancement in enzyme function mining and metabolic mechanism analysis, researchers focused on integrating heterologous lycopene pathways into non-carotenoid-producing hosts to enhance the titer and yield. *E. coli* and *S. cerevisiae*, as classic model microorganisms, have been used for large-scale synthesis of lycopene successfully [121].

Chen et al. developed a high-throughput screening method dependent on the color of lycopene. By using color as an indicator, they performed random mutations and targeted mutagenesis on the rate-limiting enzyme isopentenyl diphosphate isomerase (IDI) and obtained a triple mutant IDI^L141H/Y195F/W256C^ with 2.53-fold higher catalytic activity. Replacing the wild-type IDI with IDI^L141H/Y195F/W256C^, *E. coli* containing the complete lycopene synthetic pathway produced 1.8-fold more lycopene, exceeding 1.2 g/L [95]. In order to break the yield bottleneck, Zhou et al. performed atmospheric and room-temperature plasma (ARTP) mutagenesis and H_2_O_2_-induced adaptive laboratory evolution (ALE) to improve the supply of FPP. Inspired by the transcriptome analysis of the high-yield mutant strain, they integrated another copy of the *TmCrtE* gene to drive GGPP synthesis. In a 7 L fed-batch fermentation, the lycopene achieved the highest level, at a titer of 8.15 g/L [96].

#### 3.3.2. Steviol Glycosides

The increasing prevalence of diabetes and its harm to health promote people to pay more and more attention to low-sugar diets. The use of low-calorie sweeteners instead of sugar in the daily diet is becoming popular and important. In this scene, steviol glycosides (SGs), which are GRAS, have received widespread attention [122]. They are not only used as sweeteners but also act as nutritional enhancers in the diet of diabetic patients due to their anti-diabetic properties. SGs are a type of glycoside derivative of tetracyclic diterpenoids with various sweetness and taste. In *Stevia rebaudiana*, more than 20 types of SGs are glycosylated by only four glycosyltransferases using steviol as a backbone skeleton [97]. In order to meet the demands for low-sugar and low-calorie diets, scientists are dedicated to producing special steviol glycosides via de novo synthesis or whole-cell catalysis using microbial chassis.

Due to the complex glycosyltransferase pathway and numerous by-products, attention has been focused on exploring targeted modification of glycosyltransferases [98]. They adopted a strategy of combinatorial modular operation to assemble the stevioside de novo synthetic pathway into *S. cerevisiae*. In the precursor GGPP supply module, they overexpressed the key enzyme tHMG1 and downregulated ERG9 shunt pathways. In the steviol synthesis pathway, they used steviol as an indicator and fusion-expressed the class I diterpene synthase *At*KS and the class II diterpene synthase *Sr*CPS. In the transglycosidic pathway, they combined directed evolution and fusion expression of two glycosyltransferases to synthesize stevioside. By further enhancing the supply of UDP-glucose, the final engineered strain produced stevioside at a titer of 1149 mg/L [97]. Rebaudioside M (Reb M) provides more sweet taste than stevioside and is free of bitterness. However, the concentration is significantly lower (0.4–0.5%) than stevioside (22–62%) in *Stevia rebaudiana* [122]. Li et al. constructed a whole-cell catalytic pathway for producing Reb M from stevioside. They identified an efficient glycosyltransferase, UGT91D2, and co-expressed it with UG76G1 to convert stevioside to Reb M via Red E. In order to inhibit the hydrolysis of stevioside and strengthen the supply of sugar donor UDP-glucose, they knocked out endogenous glycoside hydrolase SCW2 and overexpressed UGP1. After introducing SIR2 to prolong the growth cycle, they obtained a titer of 12.5 g/L Reb M by using 10.0 g/L stevioside as a substrate.

### 3.4. Acting as Medicinal Health Food (MHF)

The concept of “Medicine and Food Homology” originated from traditional Chinese medicine, implying that some foods not only have nutritional value but also are capable of preventing and treating diseases. People eat plants containing active compounds to treat illnesses and enhance immunity. The Chinese Ministry of Health has listed 114 kinds of items as medicinal health foods [123]. Among them, *Pogostemon cablin*, *Herba menthae*, *Flos sophorae*, and *Ganoderma lucidum* contain significant levels of terpenoids as their respective functional factors [123,124,125]. Nevertheless, it is worth mentioning that traditional Chinese medicine may contain multiple complex components, some of which might cause adverse reactions or allergies in certain individuals. Therefore, under the condition that the efficacy of terpenoids is known or the mechanism is clear, taking the specific active factor directly is better than taking the whole herb. For instance, artemisinin has shown significant effects in treating malaria, and extracting artemisinin for treatment might be more effective than consuming the whole *Artemisia annua herb*. With the development of synthetic biology and systems biology techniques, the biosynthetic pathways of more and more terpenoids are being elucidated, making it possible to produce these valuable terpenoids using microbial cell factories. For example, researchers achieved de novo synthesis of three terpenoid compounds in *S. cerevisiae* by integrating the whole biosynthetic pathways for valerenic acid [126], oleanane-type ginsenosides [127], and (-)-borneol [52].

#### 3.4.1. Germacrene A and β-Elemene

Germacrene A with spicy and peppery scents can be used as flavor and fragrance in the food industry [11]. Moreover, it is one of the most critical intermediates in the biosynthesis of terpenoid compounds and can serve as a precursor of β-elemene and patchoulol. β-Elemene, extracted from the traditional Chinese herb *Rhizoma zedoariae* (Wen E Zhu) and *Curcuma longa* L. (Jiang Huang), is a volatile sesquiterpene and can be synthesized through intramolecular Cope rearrangement via germacrene A in vitro. It is an important flavor in Chinese Baijiu, not only giving a unique flavor to the liquor but also stimulating the consumer’s taste buds [1]. Additionally, β-elemene has also been widely used as a clinical anti-cancer drug due to its anti-tumor activity and immune protective properties [1]. Owing to their great application value, scientists attempt to heterologously synthesize germacrene A and β-elemene in microorganisms. Currently, they have been synthesized in various microorganisms, including *E. coli* [28], *S. cerevisiae* [128], *Y. lipolytica* [99], and *P. pastoris* [62].

Liu et al. identified a germacrene A synthase (*Dl*GAS) with the highest activity in *Y. lipolytica* using a bioinformatics strategy. They further enhanced the germacrene A synthetic flux by substrate-channel engineering, MVA, and acetyl-CoA supplying. The final titer of germacrene A was achieved at 39 g/L by using glucose as a carbon source [99]. Li et al. identified another germacrene synthase, LTC2, which originated from *Lactuca sativa*, that can be used in *Y. lipolytica*, and then they integrated the LTC2-expressing cassette into the genome of Po1f-Δku70. They promoted the synthesis of germacrene A through a “push-pull-restrain” strategy successively. Finally, β-elemene was produced by shifting the temperature to 250 °C, and the titer reached 5.08 g/L. This work is the first combination of engineering *Y. lipolytica* with chemical transformation [100].

#### 3.4.2. Valencene and Nootkatone

Valencene and nootkatone are sesquiterpenes derived from plants. Valencene provides an orange-like odor, while nootkatone has a grapefruit-like flavor. They are GRAS and widely used as food additives in the fragrances and flavors industry [129,130]. In addition, both valencene and nootkatone possess interesting biological activities, such as anti-cancer, anti-oxidant, and anti-inflammatory. Among them, nootkatone has been proven to be a major active component in traditional Chinese medicine *Alpinia oxyphylla* (Yi Zhi Ren). Because of their great market value, they were primarily produced by chemical synthesis. Recently, in the exploration of enzyme functions, researchers focused on biotransformation or heterologous synthesis to produce valencene and nootkatone [131].

For de novo valencene biosynthesizing, Zhu et al. introduced a valencene synthase (*Cn*VS) into an *S. cerevisiae* platform BN-91A, which was obtained through laboratory adaptive evolution and capable of utilizing mannitol as a sole carbon source. By comprehensively optimizing the metabolic network, a titer of 5.6 g/L of valencene was achieved in high-density cultivation with mannitol feeding [101]. In another research study, Ye and co-workers identified a more efficient valencene synthase (*Eg*VS) and performed site-directed mutation to increase its activity. By optimizing the biosynthetic pathway, they increased the titer of valencene. Furthermore, they adopted a GAL promoter to control URA3 expression in order to couple cell growth and biochemical pathways, and the titer of valencene reached 16.6 g/L in a high-density fermentation. Accompanying the following chemical conversion, the yield of nootkatone reached 80% [102]. The combination of biosynthesis and chemical transformation makes the industrial production of nootkatone possible. Recently, researchers have elucidated the natural nootkatone biosynthetic pathway in *A. oxyphylla* and achieved its total biosynthesis in an engineered *S. cerevisiae* strain [129]. This provides new resources and strategies for the subsequent production of nootkatone using green cell factories.

#### 3.4.3. Patchoulol

Patchoulol, which has a wooden, earthy scent, is a valuable sesquiterpene with anti-inflammatory, anti-cancer, anti-depressant, and other therapeutic properties [103]. It is widely used in the food, cosmetic, and pharmaceutical industries. Patchoulol is the major active ingredient in the Chinese medicine *Pogostemon cablin* (Huo Xiang) [123]. Since the natural yield of patchoulol is limited, efforts have been made to produce patchoulol using microorganisms.

It was reported that patchoulol synthase (PTS) is a promiscuous enzyme capable of producing at least 22 different sesquiterpene compounds with various structures, including patchoulol. Scientists have conducted research on its catalytic mechanism and product spectrum [132,133]. Zhou et al. performed a semi-rational design and obtained a PTS mutant PTS2^C415F/H454A^ with higher substrate-binding affinity and a 2.9-fold increase in the titer of patchoulol. After deleting competing pathways and strengthening the efflux pathways, the engineered *E. coli* strain produced nearly 1 g/L of patchoulol in a 5 L bioreactor [103]. In further research, they obtained another PTS mutant, PTS3mut4, with 11-fold higher than PTS2^C415F/H454A^. By deleting the cyclic AMP synthesis pathway to alleviate glucose repression and dynamically regulating glycolysis based on pyruvate biosensor, they achieved a titer of 1675 mg/L patchoulol in *E. coli* [104]. Similarly, Liu and co-workers rationally engineered PTS and obtained PTS^T404S^, which could reduce the production of the concomitant byproducts of patchoulol. Combining modifying off-pathway, enhancing the ergosterol pathway, regulating NADPH supply, and decreasing intracellular ROS level, they obtained an *S. cerevisiae* strain with a titer of 1632 mg/L patchoulol in a two-stage fermentation [105]. To our surprise, appropriately enhancing the by-pass synthesis of ergosterol not only did not reduce the flux of patchoulol synthesis but also increased cell density and accumulation of patchoulol in their research. This provides us with a new perspective for further breaking through the bottleneck of terpenoid synthesis.

## 4. Conclusions and Perspectives

Microbial cell factories have the capability to utilize cost-effective raw materials, even one-carbon materials, for the production of fine chemicals. This offers a green and sustainable platform for terpenoid production. It is essential to study natural biosynthetic pathways, analyze key enzymes and regulatory mechanisms in these pathways, and heterologously integrate them into microbial chassis cells to construct cell factories. Large-scale production via fermentation is an important strategy to meet the market demand for edible terpenoids. In recent years, scientists have been dedicated to engineering typical or non-typical microbial hosts to produce various types of terpenoids and their derivatives [10,107,134,135]. To further exploit the potential of microbial cells in edible terpenoid production, the following aspects should be taken into consideration.

First and foremost, the selection of the host is paramount. An excellent chassis can potentially double outcomes with half exertion. Selecting strains that are GRAS can, to a certain extent, avoid evaluations on food safety. For instance, *Lactococcus lactis*, a common probiotic found in fermented food, can serve as a food supplement to directly deliver active molecules to the intestine. Researchers have already constructed a pathway for lycopene production in *L. lactis* and preliminarily verified its potential in treating intestinal oxidative damage [136]. On the other hand, the selection of a precursor-rich chassis host can significantly reduce trial and error costs. Strains with abundant acetyl-CoA derivatives not only supply sufficient precursors for the terpenoid pathway but also, the metabolites derived from acyl-CoA derivatives, such as lipids, provide a hydrophobic environment for the cells conducive to the accumulation of terpenoids. Terpenoids such as monoterpenes, sesquiterpenes, and diterpenes have their respective common important intermediates in the synthetic pathway. Direct metabolic switching in existing cell factories can accelerate the strain construction process.

Secondly, reprogramming the biosynthetic pathway of terpenoids is essential. Terpenoids have diverse and complex structures. During evolution, in order to optimize the metabolic efficiency of organisms and reduce the number of unnecessary enzymes, terpenoid synthases responsible for forming the terpenoid skeleton and downstream modification enzymes often exhibit promiscuity or are bifunctional enzymes. Hence, even when a terpenoid synthesis pathway is heterologously introduced into a microbial host, there remains a potential for unexpected components. Mining enzymes with more specific functions can enhance the yield of target products and facilitate subsequent separation and purification. Nowadays, numerous strategies have been developed for mining the biosynthetic gene clusters from natural products [137]. On the other hand, to establish novel terpenoid synthesis pathways, it is crucial to identify enzymes from various species or to develop multifunctional enzymes. The rise of artificial intelligence technology enables the customization of enzymes with specific catalytic functions [138]. Moreover, preliminary advancements have been achieved in discovering and reconstructing non-typical terpenoid compound biosynthetic pathways by deep learning [139,140,141]. Considering that the formation and modification of terpenoid skeletons require multi-enzyme cascade reactions, strategies such as compartmentalization, membrane-less organelles, and protein scaffolds can potentially elevate metabolic rates [142,143]. In addition, high-throughput screening techniques offer rapid identification of strains with high-yield and high-titer. Distinct from traditional metabolite extraction followed by mass spectrometry detection, screening approaches based on biosensors [144] and growth-coupling [77] have significantly increased the screening throughput of microbial libraries.

Finally, it is noteworthy that the entire production process is suitable for commercial production. Some kinds of terpenoids with cytotoxicity could inhibit the production in the fermentation process. Adaptive evolution [145] combined with multi-omics analysis can help us quickly obtain tolerant strains and resolve tolerance mechanisms. Biocompatible and efficient extraction systems [15] and a derivatization strategy can alleviate the toxicity. Beyond the inherent impact of the product on its production, prolonged large-scale industrial production often encounters reductions in yield, titer, and productivity. Population dynamics at a single-cell resolution can provide insights into consistently maintaining the phenotype by increasing the robustness [146]. In the future, AI-assisted control algorithms integrating cellular physiology and fluid dynamics will facilitate process scaling-up optimization [147].

Terpenoids play significant roles in processing food for food quality and nutritional behavior. Theoretically, compounds with the same structure and conformation have the same physicochemical properties and physiological functions. Therefore, terpenoid compounds, whether naturally sourced or artificially biosynthesized, can be used to improve food quality. However, prior to employing the terpenoid compounds generated in cell factories as food additives, it is imperative to conduct rigorous assessments, including toxicity testing, allergenicity tests, and relevant legal and regulatory reviews. Moreover, explicitly labeling the source and dosage of terpenoid additives contributes to better support for transparency of food labels, thereby facilitating consumers to make more informed decisions.

In conclusion, the production of edible terpenoids using microbial cell factories not only requires technical developments for the chassis and fermentation processes but also the enhancement of the corresponding evaluation system and legislative support. Collaboration among industry, university, and research can expedite the commercialization of more edible terpenoid cell factories.

## Figures and Tables

**Table 1:** Production of terpenoids in prokaryotic cell factories.

Organism.	Chemicals	Strategy	Yield	Reference
*Escherichia coli*	Lycopene	Multiplex automated genome engineering (MAGE) of twenty endogenous genes to increase the flux of DXP pathway.	9 mg/g DCW	[24]
	Taxadiene	Multivariate-modular approach to metabolic-pathway engineering.	1.25 g/L	[23]
	Lycopene	Reconstitution of MVA Pathway.	40 mg/L	
	Geraniol	(1) Screening of different geranyl diphosphate synthetase (GGPS) and geraniol synthetase (GES); (2) Screening of *E. coli* hosts; (3) Using truncated leader peptides as fusion tags to improve geraniol production.	2.124 g/L	[25]
	Geraniol	(1) Introducing an alcohol acyltransferase; (2) Using another *E. coli* expressing acetyl esterase, which hydrolyzes geranyl acetate to geraniol.	13.19 g/L	[26]
	Myrcene	(1) Using a linalool dehydratase isomerase (LDI); (2) Aerobic-anaerobic two-stage fermentation.	1.25 g/L	[27]
	Germacrene A, β-Elemene	(1) Screening for germacrene A synthase; (2) RBS engineering of cyanobacteria-derived terpene synthase (NS); (3) Fusion expression of IspA and NS; (4) Rewriting the native central carbon metabolism of acetyl-coA and pyruvate; (5) High-throughput screening of NS using lycopene color as an indicator; (6) Overexpressing two membrane transporters, TolC and MsbA, to pump compounds extracellularly.	β-elemene, 3.52 g/L germacrene A, 2.13 g/L	[28]
	α-Ionone	(1) Fusing the carotenoid cleavage dioxygenase 1 (CCD1) with thioredoxin (TrxA); (2) Applying directed evolution of CCD1; (3) Over-expressing the alkyl hydroperoxide reductase (*ahp*C/F).	700 mg/L	[29]
	Dihydro-β-ionone	(1) Identifying an enoate reductase (DBR1) gene that could transform β-ionone to dihydro-β-ionone; (2) Constructed a cell-free synthesis system containing CCD1, DBR1, and glucose dehydrogenase (GDH).	13.34 mg/L	[30]
	Dihydro-β-ionone	(1) Integrating the de novo β-ionone biosynthesis pathway into *E. coli*; (2) Constructing the DBR1 module in *S. cerevisiae*; (3) Coculturing *E. coli* and *S. cerevisiae*.	27 mg/L	[31]
*Corynebacterium glutamicum*	Sesquarterpene	(1) Cloning of the novel carotenoid gene cluster to *C. glutamicum*; (2) HPLC analysis of the extracted pigment.	-	[32]
	α-Carotene	(1) Constructing a basal lycopene-producing strain; (2) Identifying a bi-functional OluLCY; (3) Integrating a heterologous MVA pathway; (4) Utilizing a multi-copy integration method to integrate OluLCY.	1.054 g/L	[33]
	Astaxanthin Astaxanthin-β-d-diglucoside	(1) Precursor pathway engineering; (2) Optimization of the fusion enzyme CrtZ~W; (3) Balancing of terminal CrtZ and CrtW expression; (4) Additional expression of a glycosyltransferase, CrtX.	103 mg/L 27 mg/L	[34]
	Coenzyme Q_10_	(1) Enhancing the supply of precursor FPP and its conversion to DPP; (2) Enhancing the supply of aromatic precursor pHBA by knocking out the side-product synthesis pathway and replacing the key synthetic enzyme with a mutant that eliminates feedback inhibition; (3) Assembling the precursor-enhancing pathway, the 10-HB condensation pathway, and subsequent modification pathways in one host.	-	[35]
*Bacillus subtilis*	Menaquinone-7 (Vitamin K2-7), β-Carotene	(1) Designing pyruvate and malonyl-CoA-responsive genetic circuits; (2) Remodeling the MEP and MVA metabolism by using the two-input-multi-output (TIMO) circuit-assisted framework; (3) Introducing AND gate to regulate heptamyl diphosphate synthase to biosynthesize MK-7 and β-carotene.	467.2 mg/L 1.9-fold increase	[36]
	Amorphadiene	(1) Fusing GFP at the N-terminus of amorphadiene synthetase (ADS) to increase the efficiency of translation; (2) Overexpressing all MEP pathway genes together with *ispA* gene; (3) Optimizing the medium.	416 mg/mL	[37]
*Synechococcus elongatus* PCC 7942	1,8-Cineole	Expressing codon-optimized cineole synthase (CnsA).	105.6 μg/g WCW	[38]
*Synechococcus* sp. PCC 7002	Pinene	Overexpression of *Abies grandis* PS (*Ag*PS).	1.525 mg/L	[39]
*Synechocystis* sp. PCC 6803	Valencene	(1) Knocking out squalene synthase and squalene hopane cyclase coding genes for the FPP branch pathway; (2) Knocking down the key enzyme CrtE in the GGPP derivative pathway; (3) Fusing IspA and valencene synthase (*Cn*VS) for co-expression.	19 mg/g DCW	[40]

**Table 2:** Production of terpenoids in eukaryotic cell factories.

Organism	Chemicals	Strategy	Yield	Reference
*Saccharomyces cerevisiae*	β-Myrcene	(1) Screening for heterologous β-myrcene synthase (MS) and truncated expression of MS; (2) Rationally designing and obtaining a high-activity mutant t*Q*iMS^D534N^; (3) Reverse fusing of t*Qi*MS^D534N^ and ERG20^WW^; (4) Deletion of the YPL062W gene and replacing yeast endogenous ERG20 with ERG20^W^; (5) A fed-batch fermentation in a 5 L bioreactor.	142.64 mg/L	[51]
	(-)-Borneol	(1) Identifying a high-efficient (-)-bornyl diphosphate synthase (*Bb*TPS3); (2) Codon-optimizing and N-terminus truncated expressing of *Bb*TPS3; (3) A fed-batch fermentation in a 5 L Bioreactor.	148.59 mg/L	[52]
	Retinol	(1) Integration of the β-carotene dioxygenase (BLH) gene into a β-carotene-producing strain; (2) Increasing the supply of precursor β-carotene and cofactor NADPH; (3) Introducing two reductases, Env9 and YbbO, in order to reduce retinal to retinol; (4) Adding butylated hydroxytoluene (BHT) to inhibit retinol oxidation.	2.479 g/L	[53]
	Carnosic acid	(1) Fuse-expressing *Sm*CPS-*Sm*KSL and BTS1-ERG20^F96C^; (2) Expressing the related modifying enzymes CYP76AK6, CYP76H24, and CYP76AH1; (3) Fused-expressing cytochrome P450 reductase (CPR) and NADPH-cytochrome B5 (Cytb5); (4) Enhancing the supply of NADPH and heme; (5) Activating the transcription of proteins folding-related genes in the ER.	75.18 mg/L	[54]
*Yarrowia lipolytica*	Lycopene	(1) Introducing IUP pathway by expressing CHK and IPK; (2) Over-expressing ACC1 and DGA1 to promote lipid accumulation; (3) Feeding exogenous palmitic acid to accumulate acetyl-CoA from β-oxidation.	4.2 g/L	[55]
	Geraniol	(1) Expressing the truncated plant-derived geraniol synthase (t*Cr*GES); (2) Integrating ERG10, HMGS, tHMG1, and IDI into the chromosome; (3) Processing parameters optimization.	1 g/L	[56]
	Sclareol	(1) Overexpressing tHMG1 and ERG20; (2) Co-expressing the key enzymes t*Ss*LPPS and *Ss*SCS of sclareol synthesis pathway; (3) Introducing *Pa*Ggpps and *Ss*Ggpps to simultaneously utilize IPP, DMAPP, and FPP to increase GGPP supply; (4) Assembling t*Ss*LPPS and *Ss*SCS into multi-enzyme complexes using protein scaffold peptide tags RIAD and RIDD to facilitate substrate trafficking.	12.9 g/L	[57]
	Squalene	(1) Knocking out Ku70 and co-expressing RAD52 and RAD59 to facilitate homologous recombination; (2) Enhancing the MVA pathway flux; (3) Increasing the supply of acetyl-CoA and NADPH; (4) Weakening the pathway for squalene degradation to ergosterol.	35 g/L	[58]
	β-Ionone	(1) Promoter engineering and rDNA-mediated multi-round iterative transformations of carotenoid cleavage dioxygenase (*Of*CCD1); (2) Co-expressing the key enzymes for xylose metabolism, xylose reductase (S*s*XR), xylitol dehydrogenase (*Ss*XDH), and endogenous xylulose kinase (XK).	-	[59]
*Ogataea* *polymorpha*	β-Farnesene	(1) Screening for β-farnesene synthase (*Aa*FS) and fusion-expressing ERG20-*Aa*FS; (2) Restoring the fatty acyl-CoA synthetase (FAA1) encoding gene in M16 and generating M16F, which blocks the accumulation of FFA; (3) Enhancing the flux of the MVA pathway and the supply of precursor acetyl-CoA; (4) Fed-batch fermentation using methanol as a carbon source.	14.7 g/L	[60]
	β-Elemene	(1) Evaluating different germacrene A synthase in *O. polymorpha*; (2) Modulating MVA pathway; (3) Enhancing the pentose phosphate pathway (PPP) to increase the supply of cofactor NADPH; (4) Fusion expressing phosphoketolase (PK) and phosphotransacetylase (PTA) to synthesize more precursor acetyl-CoA; (5) Downregulating the competitive pathway by fusing a degradation peptide CLN2^PEST^ to ERG9; (6) Fed-batch fermentation.	4.7 g/L	[61]
*Pichia pastoris*	Germacrene A	(1) Fusion-expressing protein ERG20-(PT)4P-GAS; (2) Overexpressing MVA biosynthesis pathway genes (i.e., IDI1, tHMG1, and ACS); (3) Introducing acetyl-CoA synthetase gene *ACS* to enhance the supply of acetyl-CoA; (4) Optimizing the nitrogen source in YPD.	1.9 g/L	[62]
	Santalene	(1) Integrating α-santalene synthase expression cassette TEF1p-SAS-AOX1t into the genome of STE-0; (2) Overexpressing MVA pathway key enzymes and acetyl-CoA synthase; (3) Increasing the copy number of *SAS*; (4) Optimizing the medium and fed-batch fermentation.	21.5 g/L	[63]
	Lycopene	(1) Overexpressing CrtE, CrtB, and CrtI to construct a lycopene synthetic pathway; (2) Overexpressing the endogenous HMGR and HMGS; (3) Overexpressing IDI1 from *E. coli* and increasing the copy of endogenous GGPPS gene; (4) Fed-batch fermentation.	0.714 g/L	[64]
	α-Farnesene	(1) Selecting the optimal α-farnesene synthase (*Md*AFS); (2) Strengthening the MVA biosynthetic pathway and acetyl-CoA supply pathway; (3) Introducing the IUP pathway into the peroxisome of *P. pastoris*; (4) Constructing dual-regulation strain; (5) Oleic acid and sorbitol co-feeding.	2.56 g/L	[65]
	Dammarenediol-II	Self-assembly of squalene epoxidase (ERG1) and dammarenediol synthase (*Pg*DDS) by using the PDZ domain and its corresponding ligand (PDZlig).	0.10 mg/g DCW	[66]
*Rhodosporidium toruloides*	Astaxanthin	(1) Using gamma irradiation mutagenesis to obtain a strain with a high yield of astaxanthin; (2) Using response surface methodology to optimize the large-scale culture conditions.	1.26 mg/L	[67]
	Limonene	(1) Using carotenoid-deficient *R. toruloides* as the host to block competing pathways; (2) Increasing the flux of MVA pathway; (3) Introducing an orthogonal limonene synthesis pathway based on neryl pyrophosphate (NPP) by fusion expressing neryl pyrophosphate synthase and limonene synthase *Sl*tNPPS1-*Cl*tLS1.	393.5 mg/L	[68]
	Limonene	(1) Verifying the peroxisomal targeting signals (PTSs) via fluorescence microscopy analysis; (2) Targeting MVA and limonene biosynthetic pathways to peroxisomes; (3) Dual metabolic regulation of the synthetic pathway in the cytoplasm and peroxisome.	1.05 g/L	[69]
	ent-Kaurene	(1) Selected ent-kaurene synthase from *Gibberella fujikuroi* (*Gf*KS); (2) Balancing the expression level of GGPP synthase (GGPPS) and KS; (3) Scaling up the cultivation to a 2 L bioreactor in a medium containing 75% corn stover hydrolysate.	1.4 g/L	[70]
	Amorphadiene Bisabolene	(1) Expressing the codon-optimized bisabolene synthase (BIS) and amorphadiene synthase (ADS); (2) Testing different carbon sources.	36 mg/L, 680 mg/L	[71]
	α-Bisabolene 1,8-Cineole	(1) Integrating a second BIS expression cassette; (2) Transcriptomics and proteomics analysis in the α-bisabolene producing strains; (3) Balancing the expression levels of the two terminal enzymes: GPPS and 1,8-cineole synthase to synthesize 1,8-cineole; (4) Regulating MVA pathway; (5) Optimizing the medium and fermentation process.	2.6 g/L 1.4 g/L	[72]

**Table 3:** Representative terpenoid cell factories used in food industry.

Functions	Chemicals	Host	Strategy	Yield	Reference
Flavor and fragrance	Linalool	*Pantoea ananatis*	(1) Identifying (S)-specific linalool synthase (LINS) and (R)-specific LINS; (2) Co-expressing them with mutated farnesyl diphosphate synthase (IspA*).	(S)-linalool, 5.60 g/L (R)-linalool, 3.71 g/L	[88]
	Linalool	*Saccharomyces cerevisiae*	(1) Semi-rationally designing the substrate binding pocket entrance of linalool synthase; (2) Screening for a mutant t67OMcLIS_M_^F447E^ with higher affinity and catalytic activity; (3) Targeting the linalool biosynthetic pathway to *S. cerevisiae* peroxisomes; (4) Constructing a more stable diploid engineering strain through mating.	2.6 g/L	[89]
	Myrcene	*Escherichia coli*	(1) N-terminal truncating of linalool dehydratase isomerase (LDI); (2) Optimizing the induction temperature; (3) Site-directed mutation of LDI; (4) Aerobic-anaerobic two-stage fermentation.	1.25 g/L	[27]
	Geraniol	*E. coli*	(1) Utilizing nucleoside diphosphate-x (NUDIX) instead of geraniol synthase to produce geraniol; (2) Enhancing GPP supply by optimizing MVA pathway and GPPS; (3) Fusion-expressing the efficient GPPS and NUDIX for geraniol synthesis; (4) Knocking out alcohol dehydrogenases and adopting microaerobic cultivation to avoid the conversion of geraniol to geranial; (5) Introducing the alcohol acyltransferase *Rh*AAT1 to transform geraniol into the low-toxicity geranyl acetate.	19 g/L	[90]
Biopreservatives	Limonene	*S. cerevisiae*	(1) Integrating six copy limonene synthase (tLimS) genes into genome; (2) Enhancing MVA and GPP pathways by expressing tHMG1, IDI1, and ERG20^F96W-N127W^; (3) Dynamically regulating the expression of ERG20 to restrain the competitive squalene pathway by using dCas system; (4) Increasing the supply of acetyl-CoA and NADPH by regulating the glyoxylate cycle and pentose phosphate pathway; (5) Compartmentalization engineering of MVA and limonene synthetic pathways; (6) Two-phase scale-up fermentation.	2.63 g/L	[91]
	Limonene	*Yarrowia lipolytica*	(1) Comparative transcriptomic analysis and identifying the changes in transcriptional levels of the genes in response to limonene; (2) Identifying an unknown functional protein YALI0F19492p conferring limonene tolerance; (3) Short-term adaptive laboratory evolution to improve the tolerance to and yield of limonene.	Near 8 mg/L	[92]
	Pinene	*E. coli*	(1) Identifying an effective pinene synthase *Pt*PS1^Q457L^; (2) N-terminal truncation to improve its hydrophilicity and solubility; (3) Introducing a GPPS mutant *Ag*GPPS^D90G/L175P^ to enhance the supply of precursor GPP; (4) Identifying a suitable host DH10B; (5) Adjusting fermentation conditions.	1035 mg/L	[93]
	Pinene	Cell-free system	(1) Constructing *E. coli* MEVI with an upstream MEV module and *E. coli* PINI with a downstream pinene module; (2) Constructing a cell-free system to produce pinene with MEVI and PINI; (3) Optimizing the physicochemical parameters using Plackett–Burman experimental design and the steepest ascent strategy; (4) Optimizing a single enzyme addition.	1256 mg/L	[94]
Food fortifiers and dietary supplements	Lycopene	*E. coli*	(1) Developing a high-throughput screening method dependent on the color of lycopene; (2) Obtaining a triple mutant IDI^L141H/Y195F/W256C^ by random mutations and targeted mutagenesis; (3) Replacing the wild-type IDI with IDI^L141H/Y195F/W256C^.	1.2 g/L	[95]
	Lycopene	*S. cerevisiae*	(1) Introducing phytoene dehydrogenase mutant (*Bt*CrtI^Y160F/N576S^) to increase lycopene yield; (2) Atmospheric and room-temperature plasma (ARTP) mutagenesis combining with H_2_O_2_-induced adaptive laboratory evolution (ALE); (3) Identifying the rate-limiting steps through transcriptome analysis of high-yield mutant strain; (4) Adjusting the copy number of *TmCrtE* to drive GGPP synthesis; (5) Enhancing the supply of FAD and NADPH; (6) Recovering *Ura3* marker.	8.15 g/L	[96]
	Stevioside	*S. cerevisiae*	(1) Expressing tHMG1 and down-regulating ERG9 to strengthen the supply of precursor GGPP; (2) Selecting active diterpene synthases by using the steviol yield as an indicator; (3) Fusion-expressing two diterpene synthases; (4) Combining directed evolution and fusion expression of two glycosyltransferases to synthesize stevioside; (5) Enhancing the supply of UDP-glucose by expressing key enzymes.	1149 mg/L	[97]
	Rebaudioside M	*S. cerevisiae*	(1) Knocking out endogenous glycoside hydrolase SCW2 to inhibit the hydrolysis of stevioside; (2) Identifying an efficient glycosyltransferase, UGT91D2, and co-expressing it with UG76G1 to convert stevioside to Reb M via Red E; (3) Overexpressing UGP1 to strengthen the supply of sugar donor UDP-glucose; (4) Introducing SIR2 to prolong the growth cycle; (5) Whole-cell catalyzing the production of Reb M using stevioside as the substrate.	12.5 g/L	[98]
Medicinal health food	Germacrene A	*Y. lipolytica*	(1) Identifying the most efficient germacrene A synthase (*Dl*GAS); (2) Fused expression of *Dl*GAS–ERG20; (3) Strengthening the expression of the MVA pathway through strong promoters and multi-copy integration; (4) Restraining the downstream squalene synthesis pathway by truncating the native promoter of ERG9 (P_ERG9_); (5) Increasing the acetyl-CoA supply by reducing the activity of ACC1 and promoting lipid degradation.	39 g/L	[99]
	β-Elemene	*Y. lipolytica*	(1) Identifying an efficient germacrene A synthase (LTC2) and integrating the sequence into genome; (2) Increasing the flux of MVA pathway by over-expressing the key enzymes; (3) Regulating the competition squalene synthetic pathway by substituting an inducible promoter that is feedback inhibited by ergosterol; (4) Adjusting the copy number of heterologous LTC2; (5) Fed-batch fermentation; (6) Centrifuging the broth and then heating the dodecane layer at 250 °C to obtain product β-elemene.	5.08 g/L	[100]
	Valencene	*S. cerevisiae*	(1) Performing adaptive laboratory evolution and obtaining a platform strain BN-91A, which can grow using mannitol as sole carbon source; (2) Introducing valencene pathway into BN-91A; (3) Decreasing the branch pathway by downregulating ERG9 (squalene synthase) and disrupting BTS1 (GGPPS); (4) Increasing the MVA pathway by over-expressing tHMG1 and ERG12 (mevalonate kinase); (5) Increasing copy number of valencene synthase (*Cn*VS); (6) Facilitating mannitol assimilation by over-expressing mannitol dehydrogenase (DSF1) and mannitol transporter (HXT13); (7) Enhancing NADPH supply by integrating NADH kinase (POS5) gene; (8) High cell-density fermentation.	5.6 g/L	[101]
	Valencene and nootkatone	*S. cerevisiae*	(1) Identifying another valencene synthase (*Eg*VS) with higher catalytic efficiency than *Cn*VS; (2) Semi-directed mutagenesis and obtaining an *Eg*VS mutant with the highest catalytic efficiency; (3) Optimizing the biosynthetic pathway by increasing the copy number of key enzymes and changing promoters; (4) Coupling cell growth and biochemical pathways by controlling URA3 expression with the GAL promoter; (5) High-cell-density fermentation and purifying valencene; (6) Chemically transforming valencene to nootkatone.	16.6 g/L a yield of 80%	[102]
	Patchoulol	*E. coli*	(1) Semi-rationally designing and obtaining a PTS mutant PTS2^C415F/H454A^ with higher substrate-binding affinity; (2) Expressing FPPS-(PT)_4_P-PTS2 fusion protein; (3) Deleting the competitive acetate synthetic pathway; (4) Enhancing efflux transporters MacAB and MsbA.	970.1 mg/L	[103]
	Patchoulol	*E. coli*	(1) Modifying and obtaining a PTS mutant PTS3mut4; (2) Deleting the cyclic AMP synthesis pathway to alleviate glucose repression; (3) Constructing a pyruvate biosensor and using it to regulate glycolysis pathway.	1675 mg/L	[104]
	Patchoulol	*S. cerevisiae*	(1) Expressing FPPS-PTS fusing protein and tHMG1; (2) Modifying off-pathway by deleting ROX1, YJL064W, and YPL062W; (3) Enhancing ergosterol pathway by over-expressing ERG11 and ERG24; (4) Regulating NADPH supply by over-expressing POS5; (5) Decreasing intracellular ROS by over-expressing cytoplasmic catalase (CTT1) and peroxisome catalase (CTA1); (6) Rational engineered PTS and obtained PTS^T404S^, which could reduce production of the concomitant byproducts of patchoulol; (7) High-density fermentation with Mg^2+^ addition.	1632 mg/L	[105]

## Data Availability

No new data were created or analyzed in this study. Data sharing is not applicable to this article.

## Abbreviations

The following abbreviations are used in this manuscript:

AcAc-CoA	acetoacetyl-CoA
HMG-CoA	3-hydroxy-3-methylglutaryl-CoA
MVA	mevalonate
MVA-5P	mevalonate-5-phosphate
MVA-5PP	mevalonate-5-diphosphate
DXP	1-deoxy-D-xylulose-5-phosphate
MEP	2-C-methyl-D-erythritol 4-phosphate
CDP-ME	4-diphosphocytidyl)-2-C-methyl-D-erythritol
CDP-MEP	2-phospho-4-diphosphocytidyl-2-C methyl-D-erythritol
MEC	2-C-methyl-D-erythritol 2,4-cyclodiphosphate
HMBPP	1-hydroxy-2-methyl-2-butenyl 4-diphosphate
IPP	isopentenyl pyrophosphate
DMAPP	dimethylallyl pyrophosphate
NPP	neryl diphosphate
GPP	geranyl diphosphate
FPP	farnesyl diphosphate
GGPP	geranylgeranyl diphosphate
GFPP	geranylfarnesyl diphosphate
HMGS	HMG-CoA synthase
HMGR	HMG-CoA reductase
MVK	mevalonate kinase
PMK	MVA-5P kinase
MDD	MVA-5-PP decarboxylase;
DXS	DXP synthase
DXR	DXP reductoisomerase
MCT	CD-ME cytidylyltransferase
CMK	CD-ME kinase
MDS	MEC synthase
HDS	HMBPP synthase
HDR	HMBPP reductase
NPPS	NPP synthase
GPPS	GPP synthase
FPPS	FPP synthase
GGPPS	GGPP synthase
GFPPS	GFPP synthase
MTS	monoterpene synthase
STS	sesquiterpene synthase
DTS	diterpene synthase
SS	squalene synthase
